# A novel model extended from the Bouguer-Lambert-Beer law can describe the non-linear absorbance of potassium dichromate solutions and microalgae suspensions

**DOI:** 10.3389/fbioe.2023.1116735

**Published:** 2023-03-16

**Authors:** Yen-Cheng Yeh, Bernard Haasdonk, Ulrike Schmid-Staiger, Matthias Stier, Günter E. M. Tovar

**Affiliations:** ^1^ Fraunhofer Institute for Interfacial Engineering and Biotechnology IGB, Stuttgart, Germany; ^2^ Institute of Interfacial Process Engineering and Plasma Technology, University of Stuttgart, Stuttgart, Germany; ^3^ Institute of Applied Analysis and Numerical Simulation, University of Stuttgart, Stuttgart, Germany

**Keywords:** Bouguer-Lambert-Beer law, absorption spectroscopy, microalgae, light scattering, potassium dichromate, chemical deviation

## Abstract

**Introduction:** The Bouguer-Lambert-Beer law is widely used as the fundamental equation for quantification in absorption spectroscopy. However, deviations from the Bouguer-Lambert-Beer law have also been observed, such as chemical deviation and light scattering effect. While it has been proven and shown that the Bouguer-Lambert-Beer law is valid only under very restricted limitations, there are only a few alternatives of analytical models to this law. Based on the observation in the experiments, we propose a novel model to solve the problem of chemical deviation and light scattering effect.

**Methods:** To test the proposed model, a systematic verification was conducted using potassium dichromate solutions and two types of microalgae suspensions with varying concentrations and path lengths.

**Results:** Our proposed model demonstrated excellent performance, with a correlation coefficient ( $R^{2}$ ) exceeding 0.995 for all tested materials, significantly surpassing the Bouguer-Lambert-Beer law, which had an $R^{2}$ as low as 0.94. Our results confirm that the absorbance of pure pigment solutions follows the Bouguer-Lambert-Beer law, while the microalgae suspensions do not due to the light scattering effect. We also show that this scattering effect leads to huge deviations for the commonly used linear scaling of the spectra, and we provide a better solution based on the proposed model.

**Discussion:** This work provides a powerful tool for chemical analysis and especially for the quantification of microorganisms, such as the concentration of biomass or intracellular biomolecules. Not only the high accuracy but also the simplicity of the model makes it a practical alternative to the existing Bouguer-Lambert-Beer law.

## 1 Introduction

After R. Luthar proposed the modern form of the Bouguer-Lambert-Beer (BLB) law in 1913 (Mayerhöfer et al., 2020), it has been applied as the essential equation for concentration quantification in various fields such as analytical chemistry, atmospheric physics, microbiology, etc. (Ingle & Crouch, 1988; Perkampus, 2013; Skoog et al., 2013; Skoog et al., 2017; Kirchman & Gasol, 2018; Thomas & Burgess, 2022). Although it has been referred to as a “law,” it is known that this equation is a limited law (Perkampus, 2013) and is not compatible with Maxwell’s equations (Mayerhöfer et al., 2016; Mayerhöfer et al., 2020). Meanwhile, various deviations of the BLB law have been reported and observed in different fields (Duyens, 1956; Emile LeBlanc et al., 2011; Mayerhöfer et al., 2019; Li et al., 2014; Mäntele & Deniz, 2017; Ruddy, 1994).

In this work, we focus on the chemical deviation and light scattering effect. The chemical deviation mentioned in this work refers specifically to the apparent deviation of the BLB law due to chemical equilibria. This chemical deviation occurs when the individual concentrations of the chemical forms existing in the analyte are not linear with respect to the total concentration of the analyte and the absorption coefficients of these chemical forms are not identical at the measured wavelength (Burke et al., 1972; Burke & Mavrodineanu, 1977; Ingle & Crouch, 1988). There are other types of chemical deviations, such as deviation due to high concentration (Ingle & Crouch, 1988), which are not in the scope of this work. Additionally, the light scattering effect is another well-known cause of deviation from the BLB law. The term “light scattering effect” used in this work refers to the Mie scattering by microalgae cells, and it has been shown that the BLB law can be used only in the case of single scattering (Hulst and van de Hulst, 1981; Morel & Bricaud, 1981). In the case of multiple scattering, a deviation from the BLB law occurs (Zaccanti & Bruscaglioni, 1988; Garcia-Pichel, 1994; Yun & Park, 2001; Wind & Szymanski, 2002; Mohlenhoff et al., 2005; Geider, 2013) and the radiative transfer equation (RTE) has to be solved using approximations such as Mie theory for microalgae cells (Mueller, 1973; Kitchen & Zaneveld, 1992; Lee et al., 2013; Kandilian et al., 2016; Lehmuskero et al., 2018; Ma, 2021). Compared to the BLB law, which is a simple analytical equation, solving the RTE is time-consuming and unpractical for daily application in absorption measurements.

Due to the simplicity and usefulness of the BLB law, it is still widely used despite the observed deviations. Meanwhile, different extensive models based on the BLB law have been proposed (Fernández et al., 1997; Pottier et al., 2005; Santos-Ballardo et al., 2015; Bhatt et al., 2016; Casasanta & Garra, 2018; Bozdoğan, 2022). Among these models, the so-called modified BLB law is the most widely used in near-infrared (NIR) spectroscopy, where an intercept term was added to the classical BLB law to compensate for the deviation (Brown et al., 1982; Haaland et al., 1985; Delpy et al., 1988; Sassaroli & Fantini, 2004; Kocsis et al., 2006; Scholkmann et al., 2014). In this paper, we propose a novel analytical model for non-linear absorbance by adding two exponents to the classical BLB law:

$$A=\log\left(\frac{I_{in}}{I_{out}}\right)=\varepsilon^{\prime }∙c^{\alpha }∙l^{\beta }$$ (1)

Here A is the apparent absorbance, $I_{in}$ the input light intensity, $I_{out}$ the output light intensity transmitted through the sample, $\varepsilon^{\prime }$ the effective specific absorbance, c the sample concentration, α the correction coefficient of the concentration, l the path length, β the correction coefficient of the path length. Both α and β are real positive. If α and β are equal to 1, the proposed equation is identical to the original BLB law. It should be noted that the unit of $\varepsilon^{\prime }$ is not the same as the specific absorbance in the BLB law. Instead, it depends on the real numbers α and β , since A is dimensionless.

Based on two additional correction coefficients α and β , we hypothesized that the proposed model has better descriptive performance than the BLB law. To test our hypothesis, solutions of potassium dichromate and two types of microalgae suspensions (*Phaeodactylum tricornutum* and *Chlorella vulgaris*) were prepared and analyzed. Unlike most existing experiments in absorption spectroscopy using only constant path length with varying concentration or *vice versa*, we designed systematic experiments with simultaneously varying concentration and path length. The details of our experimental design are described below.

## 2 Materials and methods

To test the performance of the proposed model and compare it with the BLB law, separate absorbance measurement series were performed with potassium dichromate solutions and microalgae suspensions. In addition, the absorbance measurements of pure pigments cholorophyll *a* and fucoxanthin were conducted for comparison with the measurements of microalgae. The potassium dichromate with a purity of greater than 99.5% was purchased from the manufacturer Merck, Germany. The two microalgae *P. tricornutum* SAG 1090-1b and *C. vulgaris* SAG 211-12 were obtained from the Culture Collection of Algae at Göttingen (SAG) and cultivated in flat-panel airlift (FPA) photobioreactors (PBR) with a volume of 6 L. Cultivations were conducted with artificial illumination by LED panels. The FPA-PBRs are equipped with Siemens PLC units to control light intensity, temperature, pH value, and substrate feeding. *Phaeodactylum tricornutum* was cultivated in a modified Mann and Myers medium (Meiser et al., 2004), maintaining the temperature at 20°C ± 1°C, pH value at 7.3 ± 0.1, and under sufficient illumination. *Chlorella vulgaris* was cultivated in a modified DSN medium (Münkel et al., 2013), keeping the temperature at 25°C ± 1°C, pH value at 6.8 ± 0.1, and under sufficient illumination. In addition, ammonium and phosphate solutions were added as nutrient sources during cultivation.

The potassium dichromate solutions and microalgae suspensions were prepared on a weight/volume basis for their concentrations. Three top-loading balances from the manufacturer Sartorius (Germany) were used for the weighings in this work, including the E2000D (readability: 1 mg), the ENTRIS224I-1S (readability: 0.1 mg), and the MSA225S-1CE-DI (readability: 0.01 mg), which are calibrated once a year. The volumetric flasks and pipettes were previously calibrated with double-distilled water (ddH_2_O) to ensure accuracy. All preparations were conducted at a room temperature of 20°C–22°C. The details of the preparations and spectral measurement procedures are described in the following.

### 2.1 Measurement setups of concentration and path length

It is important to evaluate and choose appropriate setups of concentration and path length for absorbance measurements because they will determine the dynamics of absorbance measurements. A path length of 1 cm is widely used in most of the studies for UV-Vis absorbance measurement, and the concentration range should be selected in a linear range, where the BLB law can be applied. To reexamine the validity of the BLB law and compare it with our proposed model, we chose the setups of concentration and path length within the absorbance range, that the BLB model was proven to be valid with a constant path of 1 cm. We designed systematic setups with various combinations of concentration and path length (1, 0.5, 0.2, and 0.1 cm), as shown in Tables 1, 2. For the setups of potassium dichromate (Table 1), the absorbances of the subsamples No. 1 and 5 are known to be linear with the same path length of 1 cm under various wavelengths (Burke & Mavrodineanu, 1977). Subsamples No. 1 to 4 were designed to have the same product ( c∙l) of concentration ( c ) and path length ( l ), and the concentrations of the subsamples No. 2 to 4 were calculated based on the value of c∙l of the subsample No. 1. The subsamples No. 5 to 8 were also designed with the same principle of No. 1 to 4 with smaller and proportional concentrations. According to the BLB law, samples with the same values of c∙l will have the same absorbance. With the abovementioned design, it is easy to examine if the BLB is valid, and if not, the deviation will be shown clearly among the results. The subsamples No. 9 to 11 were added additionally to generate other combinations in the known linear range. The setups of microalgae (Table 2) were designed based on the same logic as for the potassium dichromate (Table 1). The absorbances of subsamples No. 1 and 5 are known to be in a linear range under the wavelength of 750 nm for *P. tricornutum* and *C. vulgaris* based on our laboratory database, which is usually used for the estimation of their biomass concentrations. As for the setups of the pigments (Table 2), the concentrations of No. 1 and 5 were determined based on a pre-test of their absorbance spectrum.

**TABLE 1:** The setups of concentration ( c ) and path length ( l ) for the potassium dichromate solutions.

Subsample no.	1	2	3	4	5	6	7	8	9	10	11
c [g L^-1^]	0.06	0.12	0.30	0.60	0.02	0.04	0.10	0.20	0.10	0.20	0.10
l [cm]	1	0.5	0.2	0.1	1	0.5	0.2	0.1	0.5	0.2	0.1

**TABLE 2:** The setups of concentration ( c ) and path length ( l ) for the suspensions of *Phaeodactylum tricornutum* and *Chlorella vulgaris*. The pigment solutions have almost the same setups, but with smaller proportions of the concentrations: chlorophyll *a* at 10% and fucoxanthin at 2.5% of the concentrations shown in the table.

Subsample no.	1	2	3	4	5	6	7	8
c [g L^-1^]	0.08	0.16	0.40	0.80	0.04	0.08	0.20	0.40
l [cm]	1	0.5	0.2	0.1	1	0.5	0.2	0.1

### 2.2 Preparation of potassium dichromate solutions

The medium used for potassium dichromate was 0.001 N perchloric acid, which was diluted from a stock solution of perchloric acid (density = 1.53 kg L^-1^, weight percentage = 60%, Producer: Merck, Germany) with double-distilled water in a volumetric flask. The potassium dichromate powder was weighed and dissolved in 0.001 N perchloric acid in volumetric flasks to prepare 1,000 mg L^-1^ and 100 mg L^-1^ stock solutions. These stock solutions were further diluted to prepare eleven subsamples with the designed concentrations shown in Table 1. Pipettes and falcon tubes were used for these further dilutions of stock solutions. Three sets of aforementioned stock solutions of potassium dichromate were prepared separately to repeat the absorbance measurements of the subsamples.

### 2.3 Preparation of microalgae suspensions

Fresh algae cultures taken from the PBRs were first dewatered by centrifugation at 4000 RPM for 10 min and then re-suspended in 0.9% NaCl solution. This process of dewatering and re-suspension was repeated three times to keep the consistency of the sample medium for absorbance measurements. The concentration of the re-suspended sample was determined by the dry weight method. First, 5 mL of the microalgae suspension was placed on a filter paper and washed twice with 5 mL of tap water. After filtering, the filter paper containing the microalgae cells was dried at 105°C (MA35, Satorius, Germany) and weighed. The re-suspended sample was further diluted with 0.9% NaCl solution to obtain subsamples with different concentrations (see Table 2). Pipettes and falcon tubes were used for these dilutions. In the preparation of the suspensions of *P. tricornutum* and *C. vulgaris*, the suspensions were prepared individually according to the above procedures.

### 2.4 Preparation of pigment solutions

Analytical standards of two pigments, chlorophyll *a* (purity ≥97%) and fucoxanthin (purity ≥95%), were purchased from Sigma-Aldrich (Germany). The pigment solutions (chlorophyll *a* and fucoxanthin) were prepared separately as described below. First, 1 mg of the pigment powder (pre-weighted by the company) was dissolved in 1 mL of pure ethanol in the glass vial provided by the manufacturer. Then, the pigment solution with a concentration of 1 g L^-1^ was diluted stepwise in falcon tubes to obtain subsamples with the designed concentrations. The experimental setups of the pigment solutions are almost the same as those of the microalgae suspensions, but with smaller proportions of the concentrations: chlorophyll *a* at 10% and fucoxanthin at 2.5% of the concentrations shown in Table 2.

### 2.5 Absorbance measurement procedure

In this work, the absorbance measurements were conducted using a HITACHI U-2900 spectrophotometer with a spectral bandpass of 1.5 nm. The spectrophotometer was calibrated every year, including wavelength accuracy, absence of stray light, baseline deviation, and photometric linearity. To ensure the accuracy and consistency of measurements, the spectrophotometer was warmed up for at least 30 min before measuring. The measurements were performed at a room temperature of 20°C–22°C. The sample cuvettes used were quartz cuvettes (100-QS, Hellma, Germany) with four different light path lengths: 1, 0.5, 0.2, and 0.1 cm.

The spectral measurements for the different materials used in this work were conducted separately. The standard procedure for a measurement run is described as follows. First, the cuvettes were cleaned with 3 M H_2_SO_4_ for at least 20 min and rinsed 5 times with double-distilled water. After cleaning, the cuvettes were kept to air dry and then filled with the medium solvent to perform a blank measurement with the setups shown in Table 3. The cuvettes were then cleaned again with 3 M H_2_SO_4_ and double-distilled water and kept to air dry. The subsamples (Table 1 or Table 2) were then loaded into the cuvettes and sealed with parafilm to prevent evaporation. Spectral measurements were then conducted using the setups described in Table 3. For each subsample, this spectral scan was repeated three times. For potassium dichromate solutions, three measurement runs were conducted as each potassium dichromate solution was repeatedly prepared three times. For microalgae suspensions and pigment solutions, one measurement run was conducted. Each run of the aforementioned experiment was conducted on the same day.

**TABLE 3:** Experimental setups of absorbance measurements for the potassium dichromate solutions, the microalgae suspensions and the pigment solutions. N_sample_ is the number of samples prepared repeatedly. N_subsample_ is the number of subsamples diluted from the samples as described in Tables 1, 2. N_scan_ is the number of spectral scans repeated for each subsample.

Material	Medium	Wavelength	Scan speed	Interval (nm)	N_sample_	N_subsample_	N_scan_
Potassium dichromate	0.001 N perchloric acid	200–450 nm	100 nm min^-1^	0.5	3	11	3
*Phaeodactylum tricornutum*	0.9% NaCl solution	300–900 nm	400 nm min^-1^	1	1	8	3
*Chlorella vulgaris*	0.9% NaCl solution	300–900 nm	400 nm min^-1^	1	1	8	3
Chlorophyll *a*	Pure ethanol	300–900 nm	400 nm min^-1^	1	1	8	3
Fucoxanthin	Pure ethanol	300–900 nm	400 nm min^-1^	1	1	8	3

### 2.6 Statistics and data analysis

The measured data were first pre-processed to perform a regression with the BLB law and with the proposed model. The first step of pre-processing was to subtract blank measurements to remove the absorption contributed by the medium. The second step was to average the repeated measurements to obtain a single representative spectrum for each subsample array. As mentioned earlier, three sets of potassium dichromate solutions were prepared separately, and the measurement was repeated three times for each set. Therefore, each subsample of potassium dichromate solution in Table 1 contains a total of nine absorption spectra, which were averaged for later use. For microalgae suspensions and pigment solutions, each subsample in Table 2 has three absorption spectra that were also averaged. These representative spectra were then used to fit the equations of the BLB law and the proposed model, as in Eqs. 2, 3.

Bouguer-Lambert-Beer (BLB) law:

$$A_{\lambda }=A_{\lambda ,sample}-A_{\lambda ,blank}=\varepsilon_{\lambda }∙c∙l$$ (2)

Here λ is the wavelength of the input light, $A_{\lambda }$ the apparent absorbance at the wavelength of λ , $A_{\lambda ,sample}$ the apparent absorbance of the sample, $A_{\lambda ,blank}$ the apparent absorbance of the medium, $\varepsilon_{\lambda }$ the apparent specific absorbance, c the sample concentration, and l the light path length.

The proposed model:

$$A_{\lambda }=A_{\lambda ,sample}-A_{\lambda ,blank}=\varepsilon_{\lambda }^{\prime }∙c^{\alpha_{\lambda }}∙l^{\beta_{\lambda }}$$ (3)

Here λ , $A_{\lambda }$ , $A_{\lambda ,sample}$ , $A_{\lambda ,blank}$ , c and l are as above. In addition, $\varepsilon_{\lambda }^{\prime }$ is the effective specific absorbance at the wavelength of λ , $\alpha_{\lambda }$ the correction coefficient of concentration, and $\beta_{\lambda }$ the correction coefficient of the path length.

The software Matlab R2022a (MathWorks, Uunited States) was used to solve the regression problem in this work, which contains three-dimensional data points (output: $A_{\lambda }$ , inputs: c , l ). The built-in function “fit” in Matlab with the fitting method “Non-linearLeastSquares” was used to obtain the coefficients in Eqs. 2, 3. The performance of the BLB law and the proposed model was then compared using the correlation coefficient ( $R^{2}$ ) and the normalized root mean square error ( NRMSE ). The quality of the absorbance measurements was evaluated with the coefficient of variation (CV). These statistical criteria are shown in the following equations.

$$R^{2}=\frac{{\left(n\sum y_{\lambda ,i}{\hat{y}}_{\lambda ,i}-\sum y_{\lambda ,i}\sum {\hat{y}}_{\lambda ,i}\right)}^{2}}{\left(n\sum y_{\lambda ,i}-{\left(\sum y_{\lambda ,i}\right)}^{2}\right)\left(n\sum {\hat{y}}_{\lambda ,i}-{\left(\sum {\hat{y}}_{\lambda ,i}\right)}^{2}\right)}$$ (4)

Here $R^{2}$ is the correlation coefficient for each wavelength λ , where n is the sample size, $y_{\lambda ,i}$ the measured data, and ${\hat{y}}_{\lambda ,i}$ the predicted data.

$$NRMSE=\frac{1}{{\bar{y}}_{\lambda }}\sqrt{\frac{\sum_{i=1}^{n}{\left(y_{\lambda ,i}-{\hat{y}}_{\lambda ,i}\right)}^{2}}{n}}$$ (5)

Here NRMSE is the normalized root mean square error for each wavelength λ , where n , $y_{\lambda ,i}$ , and ${\hat{y}}_{\lambda ,i}$ are defined as above, and ${\bar{y}}_{\lambda }=\frac{\overset{n}{\underset{i=1}{\sum }}y_{\lambda ,i}}{n}$ is the mean of the measured data.

$$CV=\frac{\sigma_{\lambda }}{{\bar{y}}_{\lambda }}$$ (6)

Here CV is the coefficient of variation for each wavelength λ , where $\sigma_{\lambda }=\sqrt{\frac{1}{n-1}\sum_{i=1}^{n}{\left(y_{\lambda ,i}-{\bar{y}}_{\lambda }\right)}^{2}}$ is the standard deviation of the measured data and ${\bar{y}}_{\lambda }$ is defined as above.

## 3 Results and discussion

**FIGURE 1:**
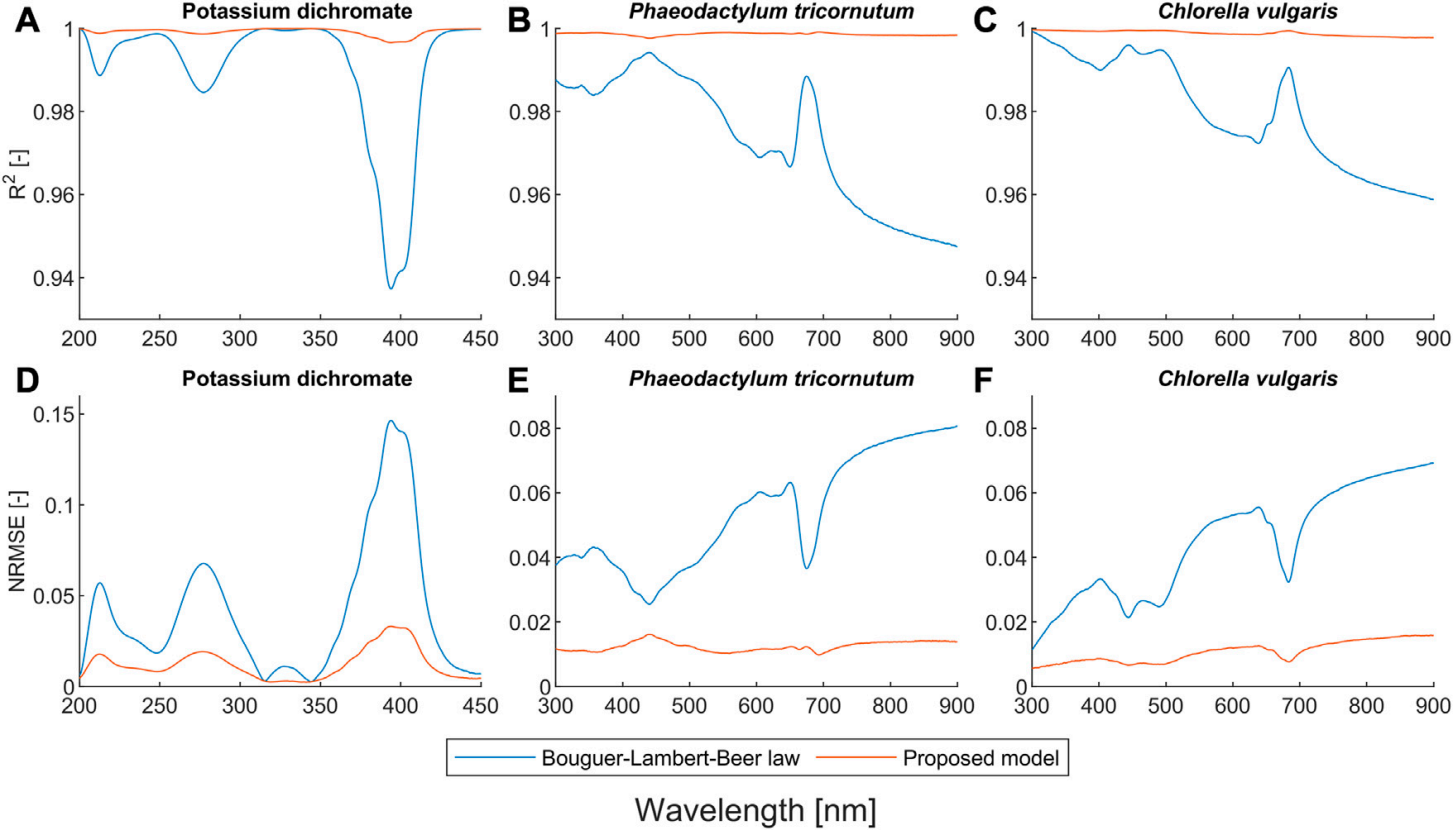
Quantitative comparison of the Bouguer-Lambert-Beer law and the proposed model in terms of $R^{2}$ and NRMSE for the potassium dichromate solutions and the microalgae suspensions.

**FIGURE 2:**
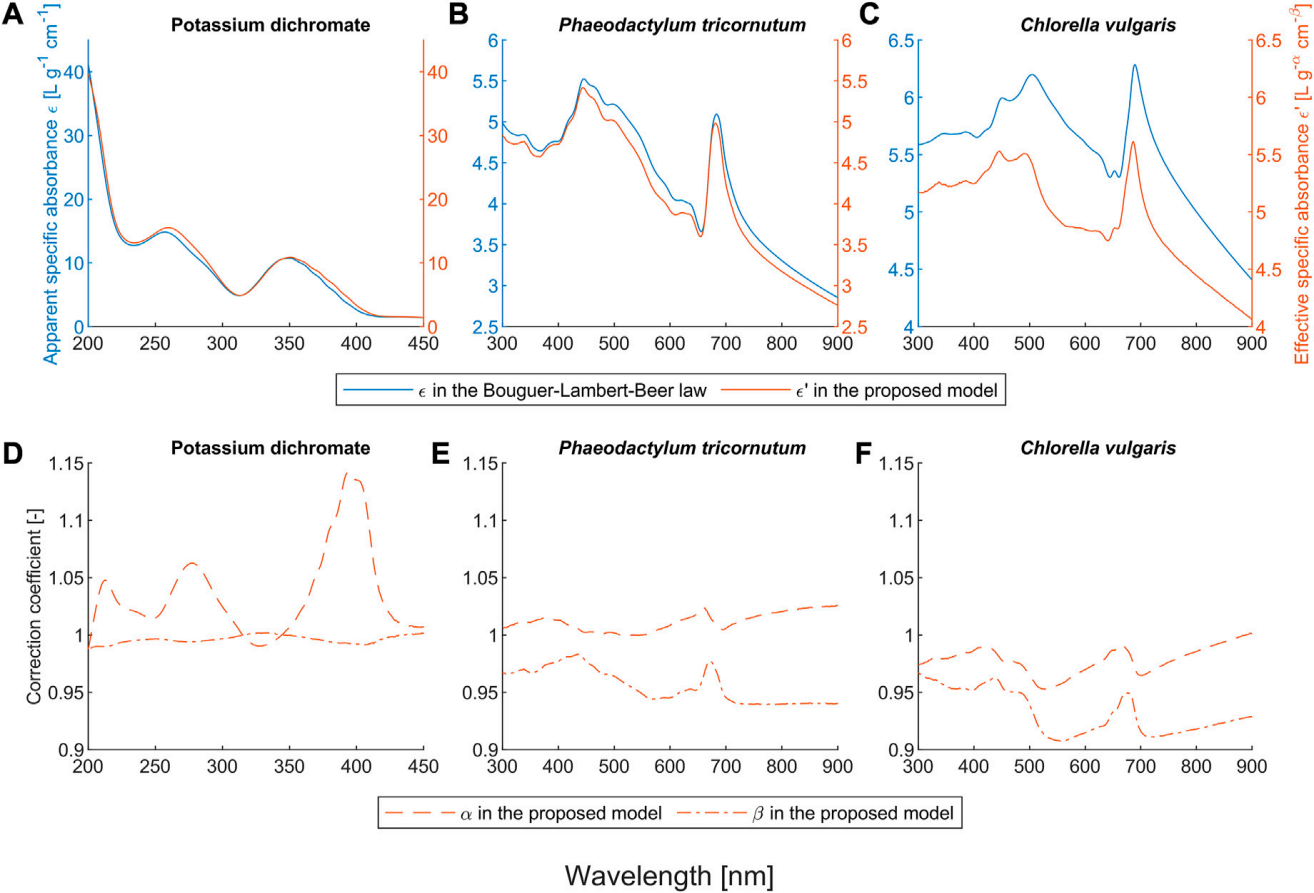
Obtained coefficients of the Bouguer-Lambert-Beer law and the proposed model for the potassium dichromate solutions and the microalgae suspensions. Here, ε denotes the apparent specific absorbance in the BLB law. For the proposed model, $\varepsilon^{\prime }$ is the effective specific absorbance, α the correction coefficient of the concentration, and β the correction coefficient of the path length.

**FIGURE 3:**
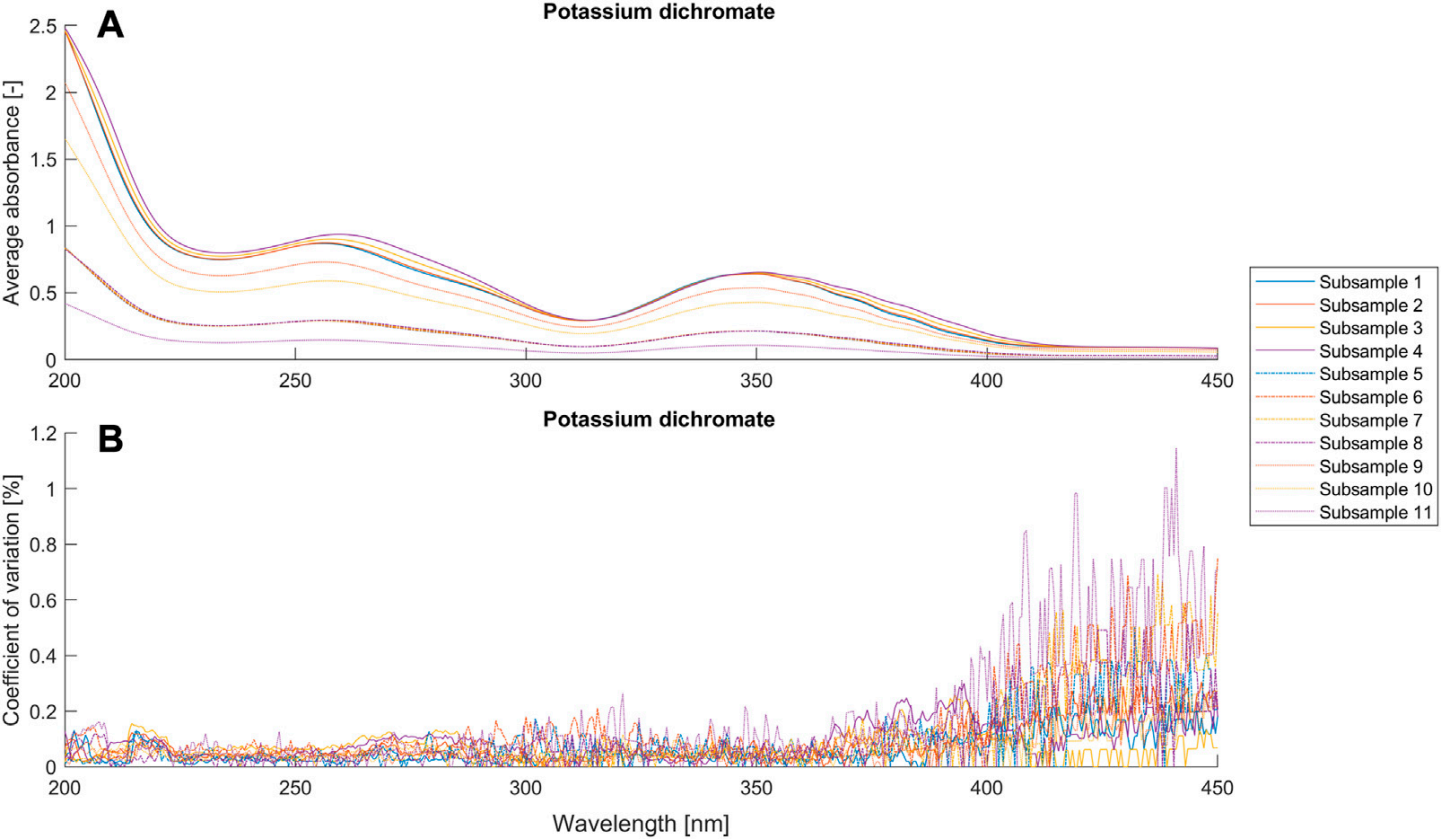
Average absorbance and coefficient of variance (*n* = 9) for different setups (Table 1) of the potassium dichromate solutions.

**FIGURE 4:**
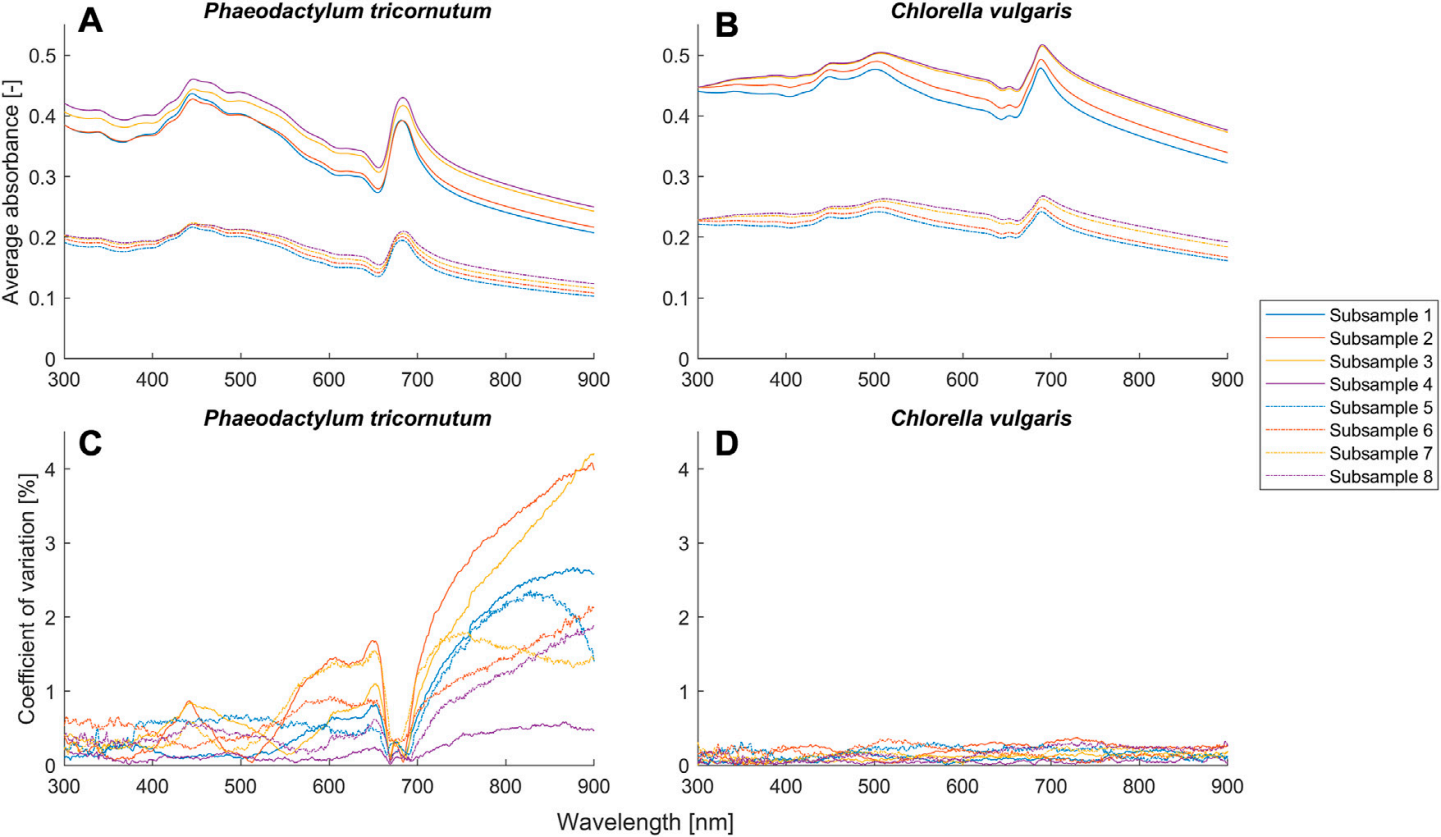
Average absorbance and coefficient of variance (*n* = 3) for different setups (Table 2) of the suspensions of *Phaeodactylum tricornutum* and *Chlorella vulgaris*.

**FIGURE 5:**
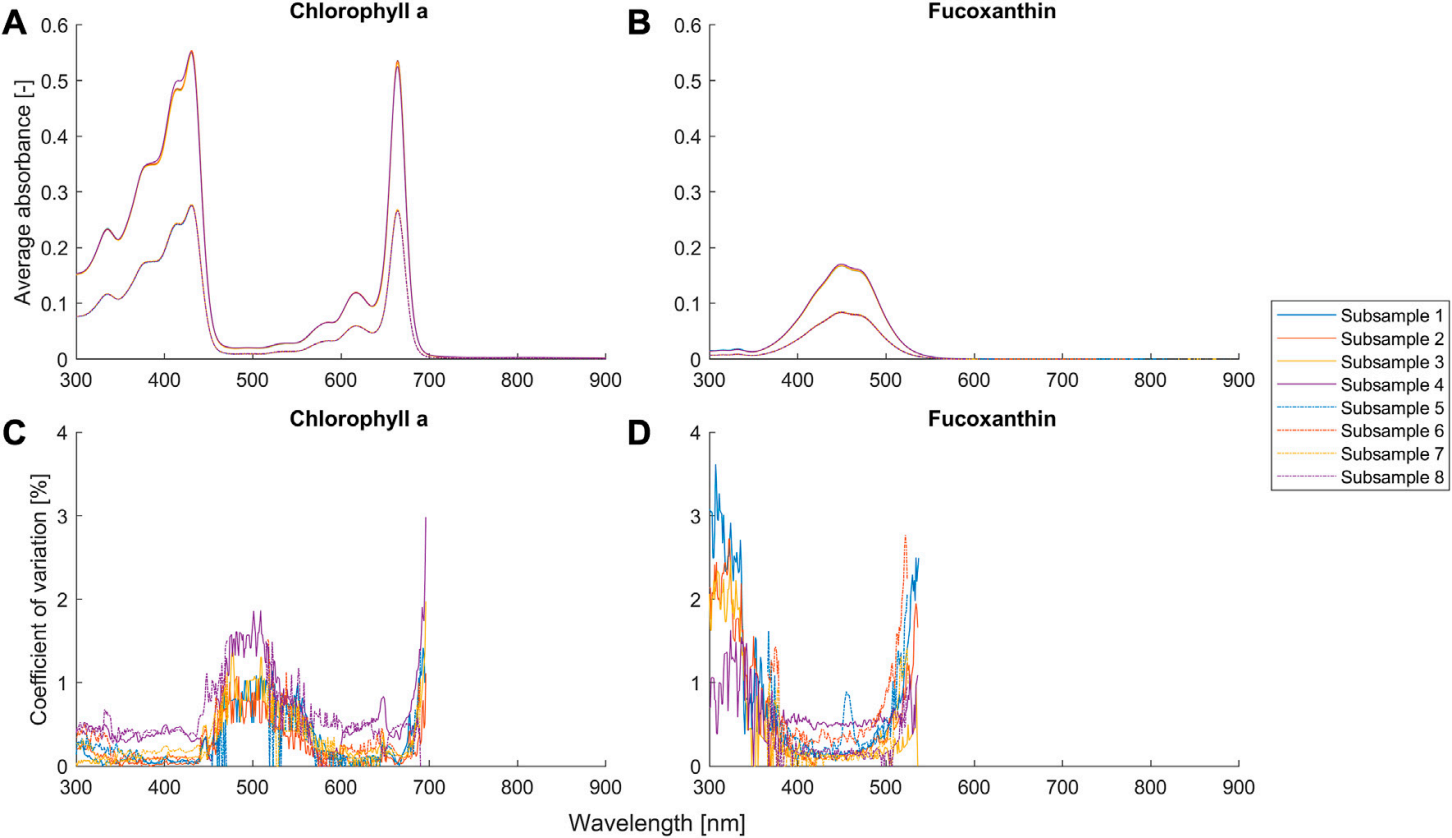
Average absorbance and coefficient of variance (*n* = 3) for different setups (Table 2) of the suspensions of chlorophyll *a* and fucoxanthin. The coefficient of variation with an average absorbance smaller than 0.01 is removed and not shown in the figures, because the signal-to-noise ratio is too small and the reflected error becomes not meaningful.

To evaluate our proposed model and compare it with the BLB law, we conducted systematic experiments with potassium dichromate solutions and two microalgae suspensions. We used the criterion $R^{2}$ and NRMSE to evaluate the performance of the models as described in the previous section. In the case of chemical deviation, the results of potassium dichromate solutions are shown in Figures 1A, D, and the obtained coefficients of the BLB law and the proposed model are shown in Figures 2A, D. For the case of light scattering effect, the results of the two microalgae are shown in Figures 1B, C, E, F, and the obtained coefficients are given in Figures 2B, C, E, F. The results of average absorbance and coefficient of variation of all the species used in this work are shown in Figures 3–5. To explore the different types of BLB law deviation, the interpretation, and discussion of the results are divided into two subsections: chemical deviation and light scattering.

### 3.1 Chemical deviation

Figures 1A, D show that the proposed model outperforms the BLB law for potassium dichromate solutions with an $R^{2}$ of more than 0.99 and an NRMSE of less than 0.03 over the entire wavebands, while the worst $R^{2}$ and NRMSE of the BLB law are around 0.94 and 0.15, respectively. As shown in Figures 2A, D, the correction coefficients of the path length ( α ) are close to one in all wavebands, confirming that the deviation of the BLB law in potassium dichromate solutions is caused by chemical equilibria, since the deviation is independent of path length and occurs only with changes in concentration (Buijs & Maurice, 1969; Burke et al., 1972; Burke & Mavrodineanu, 1977; Ingle & Crouch, 1988).

The equilibrium equation of the potassium dichromate solutions with weakly acidic media, as used in this work, is given in the equation below.

$$2HCrO_{4}^{-}⇌Cr_{2}O_{7}^{2-}+H_{2}O$$ (7)

Here, the dimerization of the monomer, $HCrO_{4}^{-}$ , forms the dimer $Cr_{2}O_{7}^{2-}$ . Apparent deviations from the BLB law are expected at wavelengths where the molar absorption coefficients of $HCrO_{4}^{-}$ and $Cr_{2}O_{7}^{2-}$ differ from each other (Burke et al., 1972; Burke & Mavrodineanu, 1977). The chemical deviation is not caused by the BLB law itself but by the non-linear chemical equilibria of the individual ions ( $HCrO_{4}^{-}$ ; $Cr_{2}O_{7}^{2-}$ ) as shown in Eq. 7. Our proposed model can describe this non-linear behavior and it is valid as long as the BLB law remains valid for the ions involved. The relationship between total analyte concentration, path length, and apparent absorbance can be expressed by a much more complicated mathematical expression, as described in Ingle & Crouch, 1988. With the proposed model, neither the equilibrium equation nor the absorption spectra of individual ions are needed to describe the absorbance characteristics, which can be used as a practical tool in analytical chemistry. Furthermore, the proposed model can identify the chemical deviation when only β is close to one and α is not, which is similar but much simpler than the polynomial methods described in (Buijs & Maurice, 1969).

Potassium dichromate is used as a reference standard to calibrate the linearity of UV-vis spectrophotometers (Burke & Mavrodineanu, 1977). This linearity is only valid within the linear range and the path length should be constant. Another common application of potassium dichromate is serving as an oxidizing agent in various reactions, where the change of chromium form can be determined by UV-visible spectroscopy and the concentration of the target analyte can be calculated. One example of this application is the chemical oxygen demand (COD) analysis, in which the wavelength 440 (435) nm is widely used for the detection of dichromate and 610 (600) nm for Cr^3+^ (Thomas et al., 2017). As shown in Figure 2D, the selection of 440 (435) is a very smart choice, because both α and β are close to one. Therefore, the BLB law is valid here, which means that the calibration of the COD determination can be extended to other setups of concentration and path length. The choice of this setup can be determined based on different conditions, for example, smaller path length for higher concentration.

### 3.2 Light scattering effect

**FIGURE 6:**
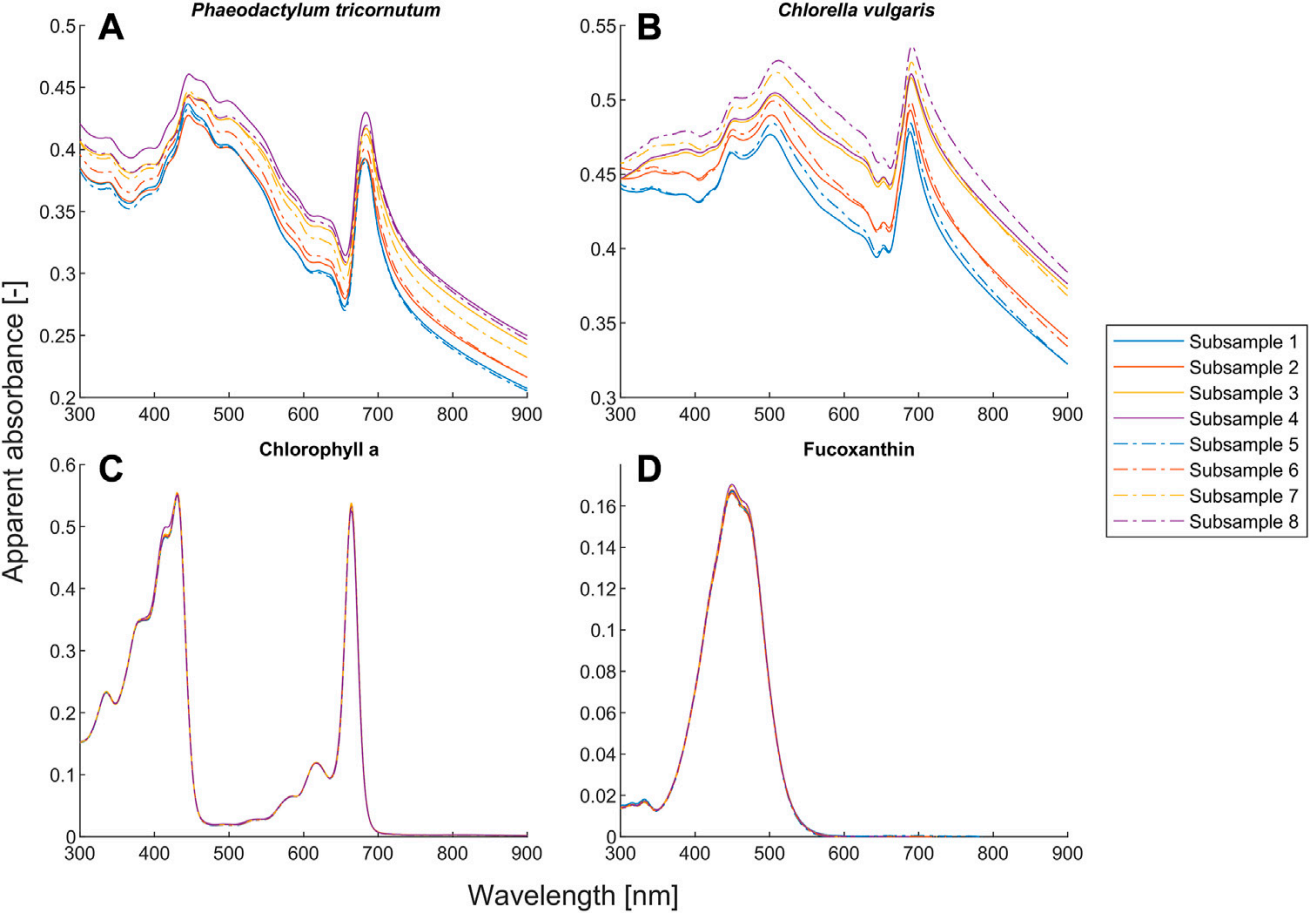
Quantitative comparison of the absorption spectra for the suspensions of *Phaeodactylum tricornutum* and *Chlorella vulgaris* and the solutions of chlorophyll *a* and fucoxanthin. The absorption spectra of subsamples No. 5 to 8 were scaled by a factor of 2 for comparison purpose. The spectra with the same path length are given in the same color. The setups of the subsamples are given in Table 2.

Our proposed model also outperforms the BLB law for scattering microalgae suspensions, as shown in Figures 1B, C, E, F. Unlike the deviation caused by chemical equilibria, the values of both α and β for microalgae suspensions mostly deviate from one (see Figures 2E, F). The deviation of the BLB law also causes the error in using linear scaling or standardization of spectra, which is commonly used for pre-processing spectral data (Craig et al., 2006; Rinnan et al., 2009; Yao & Lewis, 2010; Gholizadeh et al., 2016; Mishra et al., 2020). To demonstrate this effect, we examined the spectra of scattering microalgae suspensions and non-scattering pigment solutions, the experimental setups of which are given in Table 2. We scaled the spectra of subsamples No. 5 to 8 by a factor of 2 and plotted them together with the spectra of subsamples No. 1 to 4 in Figure 6. This is because the concentrations of subsample No. 5 to 8 are each half that of subsamples No. 1 to 4, respectively. Moreover, the product ( c∙l ) of concentration ( c ) and path length ( l ) remains the same for subsamples No. 1 to 4, and No. 5 to 8, which means that all scaled and unscaled spectra should be identical if the BLB law is valid. However, Figures 6A, B show clearly that the use of linear scaling causes deviations in spectra of scattering microalgae suspensions. Therefore, one should be very careful when applying linear scaling in the spectra of scattering suspensions. In contrast, our model can solve the aforementioned problem in different ways. First, if scaling is needed, non-linear scaling based on the proposed model should be used instead of a linear one. Second, if the goal is to compare the optical properties of different suspensions, the coefficients of the proposed model can be used instead of individual scaled spectra.

Unlike the microalgae suspensions, the results in Figures 6C, D show that the BLB law is indeed valid for pure pigment solutions because all the spectra with the same c∙l are shown to be identical. In fact, UV-Vis spectroscopy is widely used in pigment analysis of the extracts of microalgae (Wellburn, 1994; Jeffrey et al., 1997; Dere et al., 1998). This kind of pigment analysis is based on the validity of the BLB law and the additivity of absorbance. For instance, the concentration of chlorophyll *a* can be determined by solving a linear equation with absorbances at two wavelengths. These wavelengths normally correspond to the local maximums of the spectrum to maximize the signal-to-noise ratio. These maximums of the spectrum are specie and solvent dependent. Our results in Figures 6C, D show the same maximums of absorbance for chlorophyll *a* (663 nm) and fucoxanthin (450 nm) as described in the literature (Wang et al., 2018). According to the results, we can confirm that if the pigment extracts of microalgae do not contain other scattering matters, the pigment analysis can be carried out with multiple component analysis. Moreover, the calibrations of this type of pigment analysis can be extended to other setups of concentration and path length by using the BLB law. It is noteworthy that there are some other rapid pigment analyses for the determination of chlorophyll *a* and fucoxanthin with apparent absorbance of microalgae suspension without extraction. In theory, the BLB law and the additivity of absorbance cannot be applied in this type of pigment analysis based on our results. However, there are still studies of pigment analysis using the same analysis principle for microalgae suspension as used for pigment extracts (Eriksen & Iversen, 1995; Wang et al., 2018). Because of the scattering effect, this type of calibration will be disturbed by the non-linearity of absorbance and cannot be extended to other setups of concentration and path length.

A closer look at $R^{2}$ and NRMSE in Figures 1B, C, E, F reveals a trend that the stronger the absorption of the pigments, the better the accuracy of the BLB law (linear model). This can be explained by the fact that the apparent absorbance in scattering suspensions consists of two parts: absorption and scattering. The pure absorption of pigments is linear, as shown earlier, and the non-linearity is caused by the scattering effect. Therefore, the non-linear behavior of the apparent absorbance is lower when the absorption fraction of pigments is higher, and *vice versa*. This phenomenon is very evident at about 450 nm and 690 nm, which are known as the absorption bands of carotenoids and chlorophylls (Mueller, 1973; Fernández et al., 1997). For both *P. tricornutum* and *C. vulgaris*, the accuracy of the BLB law is the best and is closer to the proposed model around these pigment wavebands. On the other hand, the accuracy of the BLB law is poorest at wavelengths above 750 nm, where there is no absorption of pigments. Meanwhile, our proposed model shows excellent performance even at 900 nm, where the BLB law has the worst accuracy.

It is known that the BLB law is only valid for single scattering. A common criterion for single scattering is the optical depth τ < 0.1 (Hulst and van de Hulst, 1981; Morel & Bricaud, 1981), corresponding to an apparent absorbance A < 0.04, where τ=A⁡ln⁡10 . When the optical depth/apparent absorbance increases into the range where the scattering interactions occur between multiple cells, the BLB law can no longer be applied. In practice, the absorbance measurement of microalgae suspensions is usually outside this range, as shown by the results in Figures 6A, B. However, our proposed model is also applicable to multiple scattering when these interactions remain moderately weak. In other words, the model presented in this work extends the range of apparent absorbance with scattering effect that can be described by analytical equations. In particular, this expands the applications of absorption spectroscopy with photosynthetic microorganisms and provides a simple and powerful tool to improve the quality of spectral data preprocessing and the accuracy of calibrations such as biomass concentration. We anticipate that the model will also be very useful for the quantification of intracellular content in microorganisms.

## 4 Conclusion

In this work, we propose a powerful empirical model as an alternative to the BLB law for the quantification of absorption spectroscopy. Our results show that the proposed model is much more accurate than the BLB law in terms of $R^{2}$ and RMSE when both concentration and path length vary. The proposed model shows excellent performance to describe not only the chemical deviation in potassium dichromate solutions but also the light scattering effect in microalgae suspensions. The model greatly improves the existing BLB law and can be applied in chemical analysis and especially in the optical analysis of microorganism suspensions.

## Data Availability

The datasets presented in this study can be found in online repositories. The names of the repository/repositories and accession number(s) can be found below: https://doi.org/10.5281/zenodo.7271619.
